# Parental Misperception of Their Child's Body Weight Status Impedes the Assessment of the Child's Lifestyle Behaviors

**DOI:** 10.1155/2010/306703

**Published:** 2010-09-02

**Authors:** Marie-Eve Mathieu, Vicky Drapeau, Angelo Tremblay

**Affiliations:** ^1^Department of Kinesiology, University of Montreal, CP 6128, Succursale Centre-ville, Montreal, Quebec, Canada H3C 3J7; ^2^Research Center, CHU Sainte-Justine, Montreal, Quebec, Canada H3T 1C5; ^3^Department of Physical Education, PEPS, Laval University, Quebec City, Quebec, Canada G1K 7P4; ^4^Division of Kinesiology (PEPS), Department of Social and Preventive Medicine, Laval University, Quebec City, Quebec, Canada G1K 7P4

## Abstract

*Objectives*. To examine if distinct characteristics are associated with parental misclassification of underweight (UW), normal weight (NW), and overweight or obese (OWOB) children and the implications of misclassification on the parental evaluation of the child's lifestyle habits. *Methods*. Cross-sectional analysis (2004 sample) of the Quebec Longitudinal Study of Child Development (1998–2010) (*n* = 1,125). *Results*. 16%, 55%, and 77% of NW, UW and OWOB children were perceived inaccurately, respectively. Misperception was significantly higher in nonimmigrant parents of UW children, in highly educated parents of NW children and in NW and OWOB children with lower BMI percentiles. Erroneous body weight status identification impedes the evaluation of eating habits of all children as well as physical activity and fitness levels of UW and OWOB children. *Conclusion*. Parental misclassification of the child's body weight status and lifestyle habits constitutes an unfavorable context for healthy body weight management.

## 1. Introduction

It is well known that not all children have a healthy body weight. In North America, at least 25% of children have above normal body mass index (BMI) [1, 2]. Early interventions and treatments are needed for these children because excess weight during childhood increases the risk of being obese in adulthood and of developing adverse medical conditions [3]. Similar preoccupations also exist for underweight (UW) children. Despite the fact that they represent less than 2% of the children in developed countries, they are a group to care for because of the deleterious effects of this condition on performance, health and survival [4]. To take action, identification of overweight and obese (OWOB) and UW children, as well as key behaviors detrimental to energy balance, is of great importance. Normal weight (NW) children must also be accurately identified, as well as their lifestyle habits, from a primary prevention perspective to avoid excessive weight gain or weight loss.

In clinical settings, less than 20% of health professionals use BMI percentile charts to evaluate the body weight status of children [5]. A review of medical records reveals that only 53% of obese children are identified by clinicians [6]. Nevertheless, it turns out that clinicians are better than parents at classifying a child in the right body weight status group without relying on height and weight measurements: clinicians misclassified about 37% of children compared to about 50% for parents who evaluated their own child [7]. Many studies have documented the specific issue of inaccurate body weight status report by parents. According to these studies, misclassification can reach up to 94%, and in some cases, several factors can influence the accuracy of child body weight status perception by the parent, such as the gender and age of the child, as well as the gender, weight status and education level of the responding parent [7–26]. However, misclassification prevalence and associated factors vary considerably from one study to another, possibly due in part to population specificities. Currently, no study has been conducted in the province of Quebec (Canada), and the only study completed in Canada used a convenient sample from only one city [20]. A limitation potentially even more important is that, despite the growing number of studies in this emerging field of research, it remains unknown if factors associated with the accuracy of parental perception are the same for UW, NW, and OWOB children.

Different misclassification rates and associated factors are reported from one study to another but they all support the presence of deficient screening on the part of both the medical team and the family. This situation can result in a high number of undetected cases, and thus children who have excess or insufficient body weight are left untreated. Better recognition of a child's unhealthy body weight status by the parent is important. Lampard et al. [9] recently showed that lack of recognition of a child's unhealthy body weight by the parent warrants lower concern regarding their weight. Accurate perceptions of eating and exercise behaviors also appear important to ensure optimal body weight control. Scarce information is available on the parental perception of lifestyle habits of UW, NW, and OWOB children. It is known that a majority of mothers of UW, NW, and OWOB children perceives that their child eats not enough, enough and too much, respectively [14, 21]. Also, parents of children above NW do not perceive their child as more physically limited than nonoverweight children [17]. However, the importance of an accurate perception of the body weight status for a good evaluation of the child's lifestyle habits is unknown. 

Given the predominant role that parents play in children's health and lifestyle habits, the present study will address the following questions: (1) are factors associated with misperception of the actual body weight status of the child by his caregiver the same among all body weight status groups, and (2) is parental recognition of their child's UW, NW or OWOB status influence the evaluation of eating habits, exercise behaviors and physical capacities of the child? 

## 2. Methods

The Québec Longitudinal Study of Child Development (QLSCD 1998–2010) is conducted by the Institut de la statistique du Québec in collaboration with the Ministère de la Santé et des Services Sociaux du Québec, the Ministère de la Famille et des Aînés du Québec and the Fondation Lucie et André Chagnon. The main objective of this study is to identify and better understand the factors that contribute to social adjustment and the educational achievement of children during early childhood. A sample of children born in the Province of Québec (Canada) in 1998 has been followed since that time, along with their parents. For the purpose of this study, the 2004 sample was chosen because it was the first one with measured fitness variables. Among the 1,529 children evaluated at this time, perceived body weight status by one of their biological parents and measured height and weight were available for 1,131. Normal weight children perceived as bigger than they are (i.e., overweight) were not considered in the analysis due to their small sample size (*n* = 6; <1% of NW children). Analyses were then conducted with a subsample of 1,125 subjects. It is of note that no specific information regarding the purpose of the present study was given to the subjects and their parents. Approval from the Ethics Committee of the Institut de la statistique du Québec and consent from participants were obtained.

### 2.1. Children Measurements

Trained evaluators weighed children without shoes to the nearest 0.1 kg on a calibrated scale and measured their height with a stadiometer to the nearest 0.1 cm. Weight, height, age, and gender of each child were used to determine BMI percentiles using the 2002 Centers for Disease Control growth chart computer program [27]. The use of these growth charts were recommended for Canadian children as well as the following cutoff points: BMI percentile < 5th: UW; 5th ≤ BMI percentile < 85th: NW; BMI percentile ≥ 85th: OWOB [28]. To document fitness, the following two tests were performed under the supervision of trained evaluators: muscular endurance was assessed by counting the maximum number of sit-ups done in 30 seconds and muscle power was measured as the longest distance achieved after two attempts at the standing long jump.

### 2.2. Characteristics of the Parents

Parents were considered immigrants if they were born outside Canada and were classified as being either <35 or ≥35 years old on the day of data collection. They were categorized as having a high school diploma or less or a post high school education based on their report at the moment of data collection. 

### 2.3. Parental Perception

The interview questionnaire, available both in French and English, was administered in person to the adult who best knew the child. To assess parental perception of the child's weight status, parents answered the question “In your opinion, compared with other children the same age and for his/her height, would you say that your child…” by “Is thin/slim”, “Is of normal weight” or “Is overweight”. Children perceived accurately, leaner than they are or bigger than they are, were identified as (=), (−) and (+), respectively. Therefore, the degree of accuracy between measured body weight status and parental perception was coded as follow: UW children perceived “thin/slim”: UW(=), UW children perceived as “normal weight” or “overweight”: UW(+), NW children perceived as “normal weight”: NW(=), NW children perceived as “thin/slim”: NW(−), OWOB children perceived as “overweight”: OWOB(=), and OW/OB children perceived as “thin/slim” or “normal weight”: OWOB(−). Specific questions regarding eating behaviors, physical activity practices and fitness level are presented in Tables 2 and 3. 

### 2.4. Statistical Analysis

Pearson's chi-square tests were used to investigate whether the distribution of categorical variables differs between the groups and to document within each body weight group if the parental perception of eating behaviors differs according to their actual perception of their child's weight status. The same procedure was followed for perception of exercise behaviors and the fitness level of the child, whereas analyses of variance were used for measured fitness variables. For the purpose of the analyses, answers were grouped when needed so that less than 20% of the categories had a theoretical value below 5, a requirement for using chi-square tests. Differences among the groups for continuous variables were documented with analyses of variance. When significant differences were present in an analysis including more than two groups, Tukey test was used for post hoc comparison. Categorical values presented are *n* (% per body weight group category) and mean score (95% confidence interval) for continuous variables. Statistical analyses were performed with JMP (8.0.2) SAS Institute Inc. and the significance level was set at 0.05. 

## 3. Results

**Figure 1:**
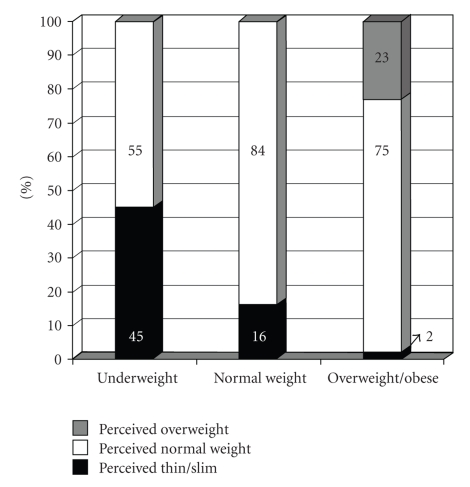
Parental perception of the body weight status of their child per measured body weight categories.

Characteristics of children (gender and age) and responding parents (gender, age, immigration status, and educational level) were similar among UW, NW, and OWOB children (data not presented). Only the BMI in percentile differed significantly between each group: 1.8 (1.4–2.1), 47.2 (45.7–48.8) and 92.7 (92.1–93.4) for UW, NW, and OWOB, respectively (*P* < .05). Figure 1 illustrates the perceived and measured weight status of the child. The accuracy of parental perception differed significantly according to the measured weight status group (*P* < .001): 84% of NW children were perceived accurately compared to only 45% of UW and 23% of OWOB children. 

Certain factors were associated with parental misperception in some but not all body weight categories (Table 1). Nonimmigrant parents were more likely to perceive that their UW child is bigger than he or she is. Parents with the highest education level were more likely to report that their NW child is thin/slim [NW(−)]. Also, a child with a lower BMI percentile within the NW or OWOB group was more likely to be perceived as leaner than he or she is. No differences in UW, NW, and OWOB children being perceived accurately were noted based on the gender and age of the child or on the age of the responding parent.

Table 2 presents results pertaining to the impact of parental misclassification of the child's body weight status on perception of eating behaviors. Children are more likely to be perceived as eating enough if they are UW(+) than if they are UW(=), while NW(−) children are more likely to be perceived by their parents as not eating enough than are NW(=) children. NW(−) and OWOB(−) children were reported to overeat less often than NW(=) and OWOB(=) children. The opposite occurs in the UW group, where being perceived as bigger [UW(+)] was related to a larger proportion of children overeating. According to their parents, children refused to eat more often if they were NW(−) than NW(=) and they refused to eat the right food more often if they were OWOB(−) than OWOB(=).

Only one difference was noted regarding the physical activity level of the child as perceived by the parent: OWOB(−) children were, compared to OWOB(=) children, two times more frequently perceived much/moderately more active than comparable children (Table 3). However, objective assessment of various physical activity practices (i.e., days per week) indicated no differences between OWOB(−) and OWOB(=) children. While UW(+) tended to be perceived as less active than UW(=) children, objective assessment of the frequency per week of unorganized sports or physical activities indicates that UW(+) are in fact more active than UW(=) children. No differences were measured between the accurate and inaccurate perception of the physical fitness of UW, NW, and OWOB children. Despite this finding, parents perceived their UW(+) children to be in worse physical fitness than did parents of UW(=) children. OWOB children classified as leaner were more likely to have a better parental evaluation of their fitness.

## 4. Discussion

Abnormal body weight status in children is a major concern for caregivers. In fact, 78% of parents reported that they would be quite or extremely concerned about their child being overweight [18] and a majority perceived being overweight as linked to future heart problems, limiting playing and exercise practices, and reducing their child's self-esteem [19]. However, parents need to be aware of their child's body weight status to worry about an unfavorable weight status and take action with body weight control [13, 17, 18]. In this regard, the results of the present study confirm what numerous studies reviewed by Towns et al. [11] indicate: parents are bad judges of their children's body weight profile. In the past, factors identified were either investigated only in OWOB children [13] or in a group composed of children of various body weight statuses [7, 8]. The present study was innovative through the identification of factors associated with misclassification specific to the child's actual body weight group: immigration status is important in the UW group, education level in the NW group and BMI percentile in the NW and OWOB groups. In fact, we showed that none of the factors affecting perception of child weight applied to all body weight groups. This study is also the first to demonstrate that parental perception of a child's lifestyle profile differs depending on whether or not the parent is aware of the child's actual body weight status. Moreover, we showed that perceived physical activity level and fitness abilities are discordant with objective assessments for many children. Globally, there is a major impact for a child to be misclassified by his or her parent that goes beyond the weight status identification alone and influences perception of key factors for body weight control.

### 4.1. Overweight and Obese Children

The case of children with excess body weight deserves special attention considering the high prevalence of OWOB in children and the health implications of this condition. If we take the proportion of unrecognized OWOB children obtained in our study (77%), which is very similar to the 73% obtained by He and Evans [20] in Ontario (Canada) and the 26% of Canadian children aged 6 to 11 who are OWOB [2], we can estimate that one out of five Canadian children in this age group is an unrecognized OWOB child. It should also be acknowledged that misclassification skewed towards a lower body weight status is higher for OWOB children with lower BMI, but a mean BMI percentile of 91.4 for OWOB(−) remains well above the 85th percentile threshold. 

Regarding the lifestyle environment and habits, it is currently known that parents of OWOB children are more likely to exert feeding restrictions [29]. However, between 43% [10] and 97% [21] of parents of OWOB children felt that their child either does not overeat or eats right/a little. One limitation of these studies is that they do not discriminate parental perception of eating behaviors based on parents' awareness of their child's body weight status. Only one study indicated that the perception of a child being overweight does not interfere with pressure to eat and restrict eating [25], but the authors did not take into account the actual body weight status of the child. To make up for this shortcoming, we investigated whether an inaccurate perception of OWOB status was associated with lack of recognition of adverse eating habits. We found that parents were significantly less likely to report that their child overeats, and that parents tend to find that the child eats too fast less often when classifying their OWOB child as leaner than he or she actually is. With evidence suggesting that eating fast leads to higher energy intake [30], this finding represents an unfavorable eating context for OWOB(−) children if they are eating fast without the parents noticing. Campbell et al. [14] also report that parents of preschool-aged children express anxiety about thinness and “picky eating” and that overweight children might be perceived as better eaters. In our study, OWOB(−) children were more frequently identified as “sometimes/often refusing to eat the right food” than were OWOB (=) children. If the same reasoning reported by Campbell et al. [14] applies to school-aged children, this evaluation could be potentially problematic for children with a positive energy balance as depicted by a BMI ≥ 85th percentile. 

As observed by Eckstein et al. [17], parents of OWOB children do not rate their child as less active or with lower physical abilities than NW children, but those aware of the OWOB status report their child less active than others [17]. This finding suggests that perception of weight status can interfere with the perception of PA and exercise behaviors. To confirm this hypothesis, two sources of information were required and available in the present study: objective questions or measures and subjective questions on physical activity and fitness levels. To this effect, we found that parents who misclassify their OWOB children [OWOB(−)] tend to rate them as more active and in better shape than parents aware of the status of their OWOB children [OWOB(=)]. This conclusion is supported by the findings of Manios et al. [12] which indicate that children seen as leaner, regardless of their actual body weight status, are perceived as more active. However, the present study also indicate that these perceptions are discordant with what parents report as the actual frequency of PA and with what is being measured for fitness. Accordingly, objective measurements or reports of physical activity and fitness levels indicate no differences between the OWOB(−) and OWOB(=) children. Thus, it is legitimate to question if parents would encourage their OWOB(−) child to increase PA and fitness levels if they are not conscious that their child is not as active or in as good shape as they think. This finding also indicates that questions used by professionals regarding exercise and fitness behaviors should avoid comparison with other children and should instead address the actual frequency and physical abilities to provide a good picture of the child's behaviors.

### 4.2. Underweight Children

The other group that has a potential energy imbalance is UW children. About half of them are perceived as bigger than they are, a result similar to that obtained by Mamum et al. [24] in a larger sample of Australian children. To our knowledge, this study is the first one to address the specific issue of weight status recognition and lifestyle assessment in UW children. In previous studies conducted with more than one body weight group, UW children were either removed because of their low number [17, 20] or grouped with NW children [14]. Assessment of their specific characteristics allowed us to determine that only in this group does one of the parental characteristics differ according to an accurate or an inaccurate evaluation. In fact, no differences were noted in the accurate perception of children based on the immigration status of the parents when all body weight groups were considered together (data not presented). However, while having immigrated to the United States earlier increased the accuracy of body weight recognition in all body weight status groups [22], we found that UW children of parents born inside the country (Canada) were more likely to being perceived as bigger than they are. No explanation is currently available to explain why this difference is present. Maybe that parents born and raised in a country and during a period where leanness is so present in the media landscape and where OWOB is so present in the society could contribute to distort the evaluation of what is a UW child. Studies that use focus groups or interview could considerably help understand why Canadian parents do not recognize the fact that their child is UW. For sure, this subgroup of UW children perceived as bigger is in a situation that can lead to the maintenance of an inadequate energy balance and may thus warrant specific consideration.

Underweight children are the other group along with OWOB children in which perception of both eating and physical activity/fitness are influenced by parental accurate perception of body weight status. When perceived as bigger than they are, UW children are more likely to eat enough and overeat according to their parents. Interestingly, UW(+) children tend to be perceived as less active [22% are identified as much or moderately more physically active compared to other children versus 41% for UW(=); *P* = .064] while they in fact take part in unorganized sports and physical activities more frequently than do UW(=) children. Therefore, children in our study or a mixed sample of children in the one by Manios et al. [12] perceived as leaner were misperceived as more active. In a similar way, UW(=) children are perceived to be in better shape than are UW(+) children, even when direct measurements reveal no difference. Globally, some parents unaware of the UW status of their child perceive that they eat too much despite a potential need for higher energy intake, and they could underestimate their child's energy expenditures versus physical activity. This combination can exacerbate the negative energy balance of these children. 

### 4.3. Normal Weight Children

Eight out of ten NW children are accurately perceived and the remaining ~20% are most likely to be perceived as leaner than they are. Children were more likely to be in the NW(−) group if their BMI is lower and their responding parent more educated. This latter finding goes against findings obtained in Italy where higher education was associated with better identification among all body weight statuses [10, 22] and in the United States where no differences in education level was present between OWOB children depending on whether or not they were accurately identified [19, 24]. The findings are, however, in line with the fact that more educated people are more inclined to give answers that conform to societal norms [31] and that the desire to be thin/slim is highly prevalent [32]. Therefore, higher social desirability could favor identification of NW children as thin/slim and subgroup analysis in this study could explain some discrepancy with previous publications.

Normal weight children might not be the group for which body weight control concerns are high but they are not protected from a shift in weight status. Genovesis et al. [10] reported that one out of four parents of NW children perceive that his or her child is not eating enough. The present study reveals that NW(−) children, despite their mean 32.4 BMI percentile, may be the ones especially targeted by parents to increase energy intake. In fact, NW(−) children are perceived to eat enough and to overeat less often than are NW(=) children. In addition, they are more likely to refuse to eat, according to their parents. Altogether, these perceptions can favor a parental predisposition to increase food intake in NW(−) children and potentially induce a positive energy balance. This conclusion is further supported by the fact that perception and objective assessments of an important factor of energy expenditure, physical activity, reveals no difference between those NW children perceived accurately or inaccurately. 

### 4.4. Limitations

Given the nature of the present study, there are limitations that need to be acknowledged. The influence of the gender of the respondent (mother or father) could not be studied because fathers were underrepresented as respondents (*n* = 18). This finding is concordant with other studies where fathers represent a low proportion of respondent [17, 19]. Normal weight children perceived as bigger than they are were also removed from the analysis due to their small number. It was also impossible to go beyond the influence of the immigration status and study the impact of the various ethnic groups regarding weight status recognition because of the small number of individuals in each group. The evaluation of eating behaviors was based only on parental perception, while fitness and PA levels were also documented objectively. It should also be recalled that conclusions obtained in this study might not apply to parent-child dyads in all countries or to children from a different age group.

### 4.5. Research and Intervention Perspectives

The difficulty associated to the accurate perception of eating, physical activity and fitness profile is a challenge for many parents. One cause that might be to considered in the present case is the fact that, on a regular basis, public health messages and publicities reinforce the link between body weight control and lifestyle behaviors (eating and exercise habits). It is possible that parents rely on something that seems easier to assess, that is, body weight status, to evaluated key components of their child energy equilibrium. An interesting area of inquiry would be to document if correcting the child body weight status perception by the parent have an impact on the evaluation of lifestyle behaviors.

Weight status identification is a simple procedure accomplished via anthropometric measurements that may be used to increase body weight status recognition. Interestingly, this awareness is desired by most parents (66%) and accepted by almost all children (96%) [29]. As a matter of fact, knowing their weight status appears to be positive for children's self-esteem, which increases in NW children and remains stable in OWOB children [29]. To increase parental awareness of the child's body weight status, family interventions appear to be necessary given that there is poor agreement between the parental recognition of the weight status of their own children and of unrelated children [22]. Also, once identified, it is essential that children with an unhealthy body weight status as well as their families are guided towards healthy and effective actions. As a matter of fact, recognition of OWOB status does not guarantee a better weight outcome for children. For example, parents of an OWOB child aware of the child's weight status were more inclined to encourage dieting, but the weight outcome in adolescents was less favorable five years later [26]. However, Grimmett et al. [29] showed that informing a family about a child's weight status in combination with providing information on healthy habits better prompts eating and physical activity changes in families. In fact, families with a NW child changed their eating and physical habits in 12% and 10% of the cases, respectively, compared to 49% and 48% in families having an OWOB child, respectively. School-based activities on recognition, evaluation and integration of healthy eating and exercise habits by the child and his family also deserve consideration for future interventions. Currently, no intervention program addresses the specific issue of body weight and lifestyle misperception. The “Healthy Mind and Healthy Body” program that promotes body weight acceptation by teenagers is potentially a good basis to the development of an intervention on bodyweight and lifestyle habits recognition since it uses a very positive approach and is design for administration in schools [33]. School-based programs appear of interest because they are the best place to reach a large number of children with body weight status and lifestyle behaviors not perceived accurately by the parent since these families won't consult for a problem they are not aware of. To target directly teenagers might be a good start for lifestyle and body weight awareness based on extrapolations by Meiser-Stedman et al. [34] made on psychological components [34]. This group showed that parents perceived less psychological impairments such as anxiety in their child following a traumatic event experienced by the child than what the child actually perceived. This raises the issue of the relative importance of body weight and lifestyle behaviors perceptions of the child and parents: does one impact more the future body weight status of the child; on who's perceptions should clinicians pay attention to correct perceptions, child or parent; and does the age of the child matters in the identification of interlocutor? For sure, the use of a multidisciplinary team (ex. nutritionist, kinesiologist and psychologist) and guidance offered to parents [35] are two key components of program designed for children body weight issues that warrant great consideration. 

Presently, the potential impact of weight status recognition is less well documented for UW children. Consequently, this lack of data reinforces the importance of developing integrated intervention and supervised programs specifically for different body weight statuses to avoid potential adverse health consequences of body weight recognition and counteract health impairments related to unfavorable body weight status. Moreover, determining the impact of accurate parental lifestyle assessments and interventions that target better recognition of these habits on body weight control of children appears to be a complementary step in this field of research.

## 5. Conclusion

Parental awareness of their child's body weight status is far from optimal, especially for UW and OWOB children. This study reveals that children and parental characteristics associated with misclassification are specific to the weight status group of the child and that these specific considerations can be used to target a specific group at higher risks of erroneous identification. Numerous differences in eating habits exist between accurately perceived and inaccurately perceived children, and this fact may suggest that parents rely on body weight status perception to appreciate the eating habits of their child. Comparison of parental perceptions and objective measurements of fitness and physical activity levels support the fact that UW and OWOB children are poorly evaluated according to the parental perception of their weight. Consequently, the familial environment of inaccurately perceived children constitutes an unfavorable context for children to adopt and maintain a healthy lifestyle, and thus to improve or maintain their body weight status. 

## Figures and Tables

**Table 1:** Characteristics of the subjects.

	Underweight			Normal weight			Overweight/obese		
	UW(=) (*n* = 37)	UW(+) (*n* = 46)	*P* value	NW(=) (*n* = 711)	NW(−) (*n* = 132)	*P* value	OW/OB(=) (*n* = 47)	OW/OB(−) (*n* = 155)	*P* value
Children									

Gender									
Male	18 (49)	24 (52)	.750	320 (45)	69 (52)	.124	19 (40)	86 (55)	.070
Female	19 (51)	22 (48)		391 (55)	63 (48)		28 (60)	69 (45)	
Age, in years	6.3 (6.2–6.4)	6.2 (6.1–6.3)	.054	6.2 (6.2-6.2)	6.2 (6.2-6.3)	.776	6.3 (6.2–6.4)	6.2 (6.2-6.3)	.383
BMI, in percentile	1.7 (1.2–2.2)	1.9 (1.4–2.4)	.567	49.7 (48.0–51.3)	32.4 (28.6–36.3)	<.001	97.2 (96.3–98.2)	91.4 (90.7–92.0)	<.001

Parent									

Age									
<35 years old	17 (46)	28 (61)	.175	329 (46)	65 (49)	.548	23 (49)	74 (48)	.886
≥35 years old	20 (54)	18 (39)		380 (54)	67 (51)		24 (51)	81 (52)	
Immigrant									
No	24 (65)	39 (85)	.035	548 (77)	103 (79)	.645	32 (68)	111 (72)	.641
Yes	13 (35)	7 (15)		160 (23)	27 (21)		15 (32)	44 (28)	
Highest diploma obtained									
High school or less	8 (22)	17 (37)	.130	242 (34)	33 (25)	.040	21 (45)	60 (39)	.464
Post-secondary	29 (78)	29 (63)		467 (66)	99 (75)		26 (55)	95 (61)	

Results are *n* (% per body weight category) for categorical and mean score (95% confidence interval) for continuous variables; BMI: body mass index; UW: underweight; NW: normal weight; OW/OB: overweight or obese; (−): perceived leaner than they are; (=): perceived accurately; (+): perceived bigger than they are. Number of subjects per category presented at the top of each column is the maximal number and is accurate for most categorical variables and all continuous variables. For precise number of subjects, calculation of subjects per category can be performed.

**Table 2:** Comparison of eating habits of children within a given body weight group perceived accurately or not.

	Underweight			Normal weight			Overweight/obese		
	UW(=)	UW(+)	*P* value	NW(=)	NW(−)	*P* value	OW/OB(=)	OW/OB(−)	*P* value
*In general, does your child…*									
*…eat enough?*									
Sometimes, rarely or never	15 (41)	5 (11)	.002	79 (11)	44 (33)	<.001	1 (2)	11 (7)	.207
Often	22 (59)	41 (89)		632 (89)	88 (67)		46 (98)	144 (93)	
*…overeat?*									
Never or rarely	37 (100)	40 (87)	.023	640 (90)	128 (97)	.010	13 (28)	114 (74)	<.001
Sometimes or often	0 (0)	6 (13)		71 (10)	4 (3)		34 (72)	41 (26)	
*…eat too fast?*									
Never or rarely	29 (78)	37 (80)	.818	559 (79)	99 (75)	.356	25 (53)	106 (67)	.056
Sometimes or often	8 (22)	9 (20)		152 (21)	33 (25)		22 (47)	49 (32)	
*…eat between meals so is not hungry at mealtime?*									
Never or rarely	16 (43)	26 (57)	.228	442 (62)	77 (58)	.406	34 (72)	104 (67)	.499
Sometimes or often	21 (57)	20 (43)		269 (38)	55 (42)		13 (28)	51 (33)	
*…eat at regular hours?*									
Never or rarely	1 (3)	1 (2)	.876	4 (1)	2 (2)	.232	1 (2)	1 (1)	.369
Sometimes or often	36 (97)	45 (98)		707 (99)	130 (98)		46 (98)	154 (99)	
*…refuse to eat?*									
Never or rarely	25 (68)	39 (85)	.064	574 (81)	89 (67)	.001	41 (87)	130 (84)	.575
Sometimes or often	12 (32)	7 (15)		137 (19)	43 (33)		6 (13)	25 (16)	
*…refuse to eat the right food?*									
Never or rarely	15 (41)	17 (37)	.739	264 (37)	43 (33)	.318	28 (60)	62 (40)	.018
Sometimes or often	22 (59)	29 (63)		447 (63)	89 (67)		19 (40)	93 (60)	

Values are *n* (%) per category of parental perception; UW: underweight; NW: normal weight; OW/OB: overweight or obese; (−): perceived leaner than they are; (=): perceived accurately; (+): perceived bigger than they are.

**Table 3:** Comparison of physical activity level and fitness of children within a given body weight group perceived accurately or not.

	Underweight			Normal weight			Overweight/obese		
	UW(=)	UW(+)	*P* value	NW(=)	NW(−)	*P* value	OW/OB(=)	OW/OB(−)	*P* value
*In your opinion, how physically active is your child compared to other children the same age and sex?* ^†^									
Much or moderately more	15 (41)	10 (22)	.064	235 (33)	52 (39)	.161	7 (15)	58 (37)	.004
Equally, moderately or much less	22 (59)	36 (78)		475 (67)	80 (61)		40 (85)	97 (63)	

*In the last 12 months, outside of school hours, how often has your child taken part in sports with a coach or instructor (except dance or gymnastics)?* ^§^									
Most days or a few times a week	4 (11)	7 (15)	.556	108 (15)	18 (14)	.646	9 (19)	23 (15)	.478
About once a week or less	33 (89)	39 (85)		603 (85)	114 (86)		38 (81)	132 (85)	

*In the last 12 months, outside of school hours, how often has your child taken lessons or instruction in other organized physical activities with a coach or instructor such as dance, gymnastics, martial arts or circus arts?* ^§^									
Most days or a few times a week	4 (11)	4 (9)	.746	55 (8)	9 (7)	.715	5 (11)	11 (7)	.431
About once a week or less	33 (89)	42 (91)		656 (92)	123 (93)		42 (89)	144 (93)	

*In the last 12 months, outside of school hours, how often has your child taken part in unorganized sports or physical activities without a coach or instructors?* ^§^									
Most days or a few times a week	19 (51)	34 (74)	.034	478 (67)	81 (61)	.190	27 (57)	99 (64)	.426
About once a week or less	18 (49)	12 (26)		233 (33)	51 (39)		20 (43)	56 (36)	

*Compared to other children of your child's age and sex, how do you consider the physical fitness level of your child?* ^†^									
Much or moderately more	12 (32)	6 (13)	.033	231 (33)	49 (67)	.304	9 (19)	54 (35)	.042
Equally, moderately or much less	25 (68)	40 (87)		479 (67)	83 (63)		38 (81)	101 (65)	

*Measured physical fitness* ^§^									
Sit ups	7.3 (5.6–9.1) *n* = 36	6.4 (5.6–9.1) *n* = 44	.370	7.4 (7.1–7.8) *n* = 674	6.7 (5.7–7.6) *n* = 121	.105	5.2 (3.8–6.5) *n* = 45	6.4 (5.6–7.1) *n* = 137	.128
Long jump	88.7 (80.4–96.9) *n* = 35	87.5 (80.9–94.1) *n* = 46	.823	96.3 (94.5–98.1) *n* = 691	94.6 (89.9–99.2) *n* = 125	.474	84.7 (77.9–91.4) *n* = 46	92.0 (87.8–96.3) *n* = 144	.084

Categorical values are *n* (% per category of parental perception, i.e., −, = or +); continuous values are mean score (95% confidence interval) UW: underweight; NW: normal weight; OW/OB: overweight or obese; (−): perceived leaner than they are; (=): perceived accurately; (+): perceived bigger than they are. ^†^: subjective assessment; ^§^: objective assessment.
